# Rapid sequencing‐based diagnosis of infectious bacterial species from meningitis patients in Zambia

**DOI:** 10.1002/cti2.1087

**Published:** 2019-11-05

**Authors:** So Nakagawa, Shigeaki Inoue, Kirill Kryukov, Junya Yamagishi, Ayumu Ohno, Kyoko Hayashida, Ruth Nakazwe, Mox Kalumbi, Darlington Mwenya, Nana Asami, Chihiro Sugimoto, Mable M Mutengo, Tadashi Imanishi

**Affiliations:** ^1^ Department of Molecular Life Science Tokai University School of Medicine Isehara Japan; ^2^ Micro/Nano Technology Center Tokai University Hiratsuka Japan; ^3^ Department of Emergency and Critical Care Medicine Tokai University School of Medicine Isehara Japan; ^4^ Department of Disaster and Emergency Medicine Kobe University Graduate School of Medicine Kobe Japan; ^5^ Research Center for Zoonosis Control Hokkaido University Sapporo Japan; ^6^ Global Station for Zoonosis Control GI‐CoRE Hokkaido University Sapporo Japan; ^7^ Department of Pathology and Microbiology University Teaching Hospital Lusaka Zambia; ^8^ Institute of Basic and Biomedical Sciences Levy Mwanawasa Medical University Lusaka Zambia

**Keywords:** meningitis, meta 16S rRNA sequencing, microbiome, nanopore sequencing, rapid diagnosis

## Abstract

**Objectives:**

We have developed a portable system for the rapid determination of bacterial composition for the diagnosis of infectious diseases. Our system comprises of a nanopore technology‐based sequencer, MinION, and two laptop computers. To examine the accuracy and time efficiency of our system, we provided a proof‐of‐concept for the detection of the causative bacteria of 11 meningitis patients in Zambia.

**Methods:**

We extracted DNA from cerebrospinal fluid samples of each patient and amplified the 16S rRNA gene regions. The sequencing library was prepared, and the sequenced reads were simultaneously processed for bacterial composition determination using the minimap2 software and the representative prokaryote genomes.

**Results:**

The sequencing results of four of the six culture‐positive samples were consistent with those of conventional culture‐based methods. The dominant bacterial species in each of these samples were identified from the sequencing data within only 3 min. Although the major bacterial species were also detected from the other two culture‐positive samples and five culture‐negative samples, their presence could not be confirmed. Moreover, as a whole, although the number of sequencing reads obtained within a short sequencing run was small, there was no change in the major bacterial species over time with prolonged sequencing. In addition, the processing time strongly correlated with the number of sequencing reads used for the analysis.

**Conclusion:**

Our results suggest that time‐effective analysis could be achieved by determining the number of sequencing reads required for the rapid diagnosis of infectious bacterial species depending on the complexity of bacterial species in a sample.

## Graphical abstract

This study used a portable system for rapid bacterial composition determination. The right PC connected to the MinION DNA sequencer obtains the DNA sequence, while the left PC analyzed the DNA sequence simultaneously.

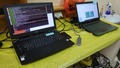

## Introduction

Advances in DNA sequencing technology have now enabled obtaining real‐time DNA sequencing data using the nanopore‐based sequencer MinION (Oxford Nanopore Technologies, Oxford, UK).^1^ Recently, remarkable performances of the MinION system have been reported for rapid bacterial identification based on sequencing of full‐length 16S rRNA gene amplicons.^2^, ^3^, ^4^, ^5^, ^6^, ^7^, ^8^ Using this sequencer in tandem with two laptop computers, we have developed a portable and rapid bacterial composition analysis system for the on‐site diagnosis of infectious diseases.^2^, ^3^, ^4^ Although this portable system could successfully determine the bacterial composition by sequencing 16S rRNA genes, there are two major challenges that need to be overcome for improved utility: the quality of DNA sequencing and the speed of sequencing searches.

The first challenge is that the quality of nanopore sequencing was found to be considerably lower, by about 85% for 1D sequencing^9^ than that obtained using more common next‐generation sequencers such as Ion PGM (Thermo Fisher Scientific, MA, USA). However, with the advantage of long‐read sequencing, MinION can detect bacterial species based on the full‐length 16S rRNA gene, which improves the accuracy of species detection.^2^, ^3^, ^4^, ^5^, ^6^, ^7^, ^8^ Moreover, nanopore sequencing technology is continuously being updated, resulting in improvements in both the quality and quantity of the output sequencing data. Therefore, the newer versions of MinION flow cells with updated library preparation kits should help to improve the quality issue. In addition, multiplex sequencing is now possible using DNA barcoding technology for 1D sequencing, which helps to reduce the cost of sequencing.

Secondly, we previously used two computer programs for the identification of bacterial species in a given sample: BLASTN^10^ and Centrifuge^11^. On the one hand, BLASTN is a relatively sensitive sequencing similarity tool and is suitable for MinION reads, but requires high computational power; thus, it takes a long time for regular laptop PCs to process the huge amount of sequence data.^4^ On the other hand, Centrifuge is superior in terms of processing time, but its accuracy is considerably lower than that of BLASTN.^4^ Recently, various computational programs have been released to handle nanopore sequencing data, including minimap^12^, minimap2^13^ and minialign^14^. In particular, minimap2 can handle compressed (gzipped) fasta data as the database, which is convenient for use with a laptop PC in terms of data capacity. Therefore, considering the speed and accuracy of sequencing similarity searches, we applied minimap2 for bacterial detection in our portable system.

With these improvements, we recently updated our rapid diagnosis system for bacterial infection^3^, ^4^. Using this system, we have conducted sequencing analyses of mock bacteria samples^3^ and aspiration pneumonia^4^ to evaluate the DNA preparation methods^3^ and identify a causative agent^4^, respectively. We successfully identified bacterial species in both studies. However, the accuracy, as well as time efficiency, was not evaluated between the previous and current systems, in particular for the performance of BLASTN and minimap2 software. More importantly, these studies were conducted in Japan, but this portable sequencing‐based system for the identification of bacterial species can work in resource‐poor countries as well. Therefore, in this study, we brought this system developed in Japan to Zambia and used it to identify the causal bacteria in meningitis patients in Zambia.

Meningitis is an infectious disease of the central nervous system caused by a bacterial or viral infection, resulting in significant morbidity and mortality, often leading to severe consequences.^15^ Approximately 4100 cases of bacterial meningitis are diagnosed in the United States each year, 500 of which are fatal.^16^ The traditional diagnostic workup of meningitis consists of neuroimaging, cerebrospinal fluid analysis (cell counts, Gram staining, biochemical tests for glucose and protein, and cultures) and blood cultures.^17^ The diagnosis of nosocomial bacterial meningitis is made on the basis of the results of a cerebrospinal fluid culture; thus, both aerobic and anaerobic culturing techniques are obligatory. However, these cultures require prolonged incubation periods before confirmation of a negative result can be made, and some results may be negative in infected patients who received previous anti‐microbial therapy.^17^ Accordingly, securing a final diagnosis can take weeks or months of testing, and many cases will remain unsolved, necessitating empirical treatment approaches that may be ineffective or even harmful to the patient.^18^

**Figure 1:**
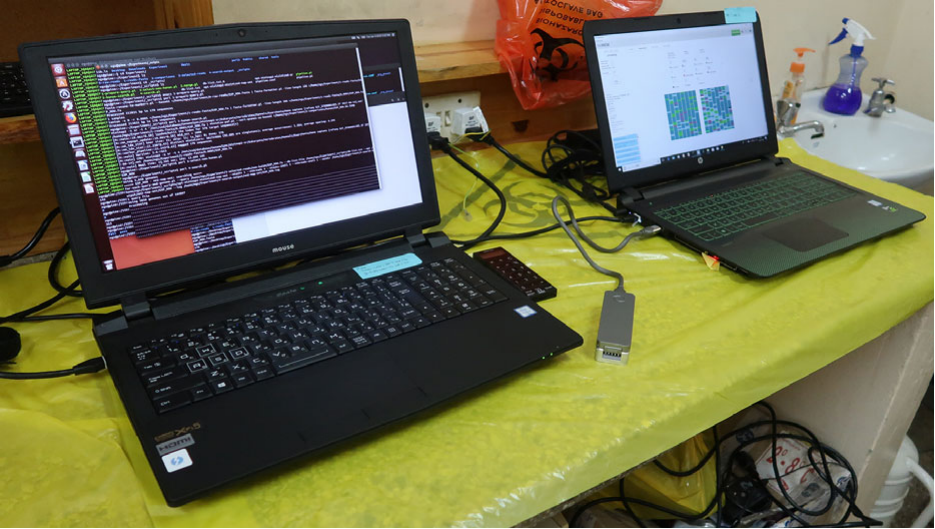
Our portable system for rapid bacterial composition determination. The right PC is connected to the MinION sequencer and handles the sequencing data, while the left PC processes the incoming data simultaneously via a LAN cable. All experiments, including sequencing and analysis, were performed at the University Teaching Hospital in Zambia.

Therefore, the critical step in the improvement of therapeutic effectiveness in meningitis is the accurate identification of the causative agents, which can ensure appropriate treatment decisions.^19^ Indeed, metagenomic next‐generation sequencing of the cerebrospinal fluid or brain tissue can screen for nearly all potential central nervous system infectious agents and can also identify novel or unexpected pathogens.^20^, ^21^ In this study, we brought our updated portable sequencing‐based system developed in Japan to Zambia for the rapid diagnosis of bacterial species in a given DNA sample (Figure 1). We then conducted 16S rRNA gene amplicon sequencing analyses of samples from spinal meningitis patients in Zambia and compared the performance of the system to the results obtained with conventional culture‐based methods.

## Results

**Figure 2:**
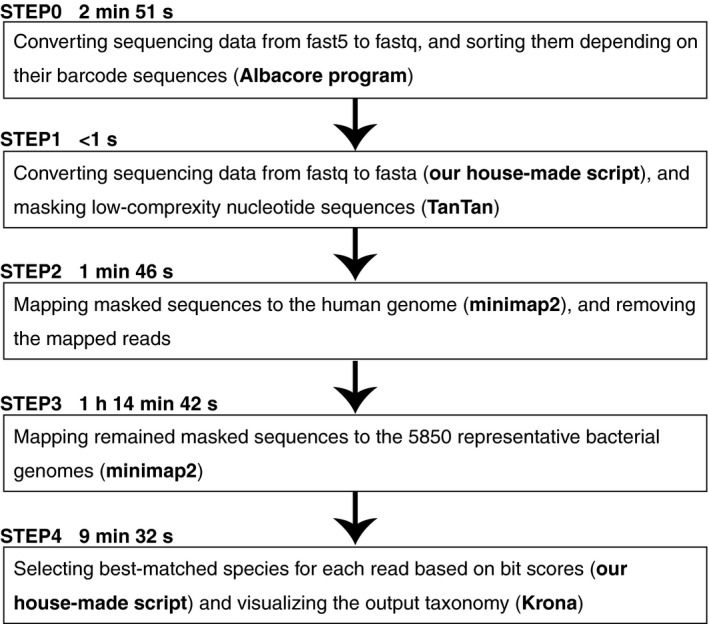
Schematic outline of the data analysis procedure. The processing time of 3‐min sequencing data is shown for each step. The detail processing time for each sample is summarised in Table 3.

Spinal fluid samples were obtained from 11 meningitis patients at the University Teaching Hospital in Zambia, where all experiments were performed except for some downstream computational analyses. The DNA was extracted from each sample, and 16S rRNA amplicon libraries were constructed. Sequencing was performed on the MinION Mk1b system without an Internet connection (see the Methods for details). As a result, 60,671 reads were obtained through 18‐hour (h) sequencing, 54,442 (89.7%) of which were sorted into 12 samples based on barcodes designed for SQK‐RAB201. The average length of sequencing reads with barcodes was 1407 base pairs (bp), which nearly corresponds to the full length of the 16S rRNA gene. For each sample, we conducted minimap2^13^ searches of all MinION reads against 5,850 representative bacterial species genomes (see Supplementary figure 1 and Supplementary tables 1–3) and predicted bacterial species in each sample at different calculation times (Figure 2). The details of the bacterial species identified at > 10% of the proportion of the entire reads at 18 h are shown in Table 1.

**Table 1:** Summary of sequencing analyses

Sample ID/Read count	Culture‐based results	Predicted bacterial species using minimap2*	Predicted bacterial species using BLASTN*
#1/19	*Escherichia coli*	*S. mitis* (35%), *G. haemolysans* (18%), *K. pneumoniae* subsp. *pneumoniae* (12%)	*S. mitis* (41%)
#2/1,050	*Enterobacter*	*E. hormaechei* subsp. *steigerwatti* (71%), *K. pneumoniae* subsp. *pneumoniae* (10%)	*E. vulneris* (16%), *K. pneumoniae* subsp*. pneumoniae* (11%), *S. enterica* subsp. *enterica* (10%)
#3/4,764	*Enterobacter*	*E. hormaechei* subsp. *steigerwatti* (63%), *A. indicus* (13%)	*E. vulneris* (18%), *A. indicus* (14%), *S. enterica* subsp. *enterica* (11%)
#4/35,557	*Pseudomonas aeruginosa*	*P. aeruginosa* (95%)	*P. aeruginosa* (99%)
#5/774	*Klebsiella pneumoniae*	*K. pneumoniae* subsp. *pneumoniae* (92%)	*K. pneumoniae* subsp. *pneumoniae* (94%)
#6/4,259	*Enterobacter*	*K. pneumoniae* subsp. *pneumoniae* (93%)	*K. pneumoniae* subsp. *pneumoniae* (96%)
#7/370	Negative	*B. thuringiensis* (40%), *B. manliponensis* (22%), *B. anthracis* (17%)	*B. cereus* group (92%)
#8/1,600	Negative	*O. turbata* (26%), *C. bogoriensis* (18%), *C. cellulans* (10%), *P. marina* (10%)	*O. turbata* (41%)
#9/69	Negative	*S. maltophilia* (57%), *D. acidovorans* (29%), *S. chelatiphaga* (14%)	–
#10/2,270	Negative	*M. chocolatum* (54%), *S. hofmannii* (17%), *P. urativorans* (12%)	*M. chocolatum* (56%)
#11/3,705	Negative	*S. pneumoniae* (39%), *H. influenzae* (20%), *S. mitis* (13%), *C. acnes* (11%)	–
#12/5	(water; negative control)	*C. acnes* (75%), *D. proteolyticus* (25%)	*C. acnes* (50%)

* Only bacterial species accounting for > 10% of the total at 18 h of sequencing are listed.

Sequencing results for four of the six culture‐positive samples were consistent with those of conventional culture‐based methods: *Enterobacter hormaechei* subsp. *steigerwatti* (71%), *Enterobacter hormaechei* subsp*. steigerwatti* (63%), *Pseudomonas aeruginosa* (95%) and *Klebsiella pneumoniae* subsp*. pneumoniae* (92%) were detected as major bacterial species for Samples #2, #3, #4 and #5, respectively. Since we sequenced almost the entire region of 16S rRNA genes, we could detect candidates of causative bacteria at the species level for each sample (Table 1), which is quite difficult to achieve using the culture‐based method. However, the other two culture‐positive samples (i.e. Samples #1 and #6) showed different potential causative bacteria compared to the results obtained by the culture‐based method. In addition, for Samples #2 and #3, other bacterial species were also detected with reads > 10%: *Klebsiella pneumoniae* subsp*. pneumoniae* (10%) for Sample #2 and *Acinetobacter indicus* (13%) for Sample #3, indicating that multiple bacteria, rather than single, may be involved in the infection in these samples.

We also detected bacterial species in the five culture‐negative samples (Table 1). For Sample #10, with 2,262 matched reads, *Microbacterium chocolatum* (54%) was detected as the major bacterial species, followed by *Scytonema hofmannii* (17%) and *Psychrobacter urativorans* (12%). For Sample #9, *Stenotrophomonas maltophilia* (57%) was detected as the major bacterial species, although this result was based on only seven mapped reads. For the other three samples, no bacterial species were detected with > 50% matched reads. However, for Sample #7, three *Bacillus* species [*Bacillus thuringiensis* (40%), *Bacillus manliponensis* (22%) and *Bacillus anthracis* (17%)] were identified, even though their 16S rRNA sequences are almost identical (97.9%). Indeed, more than 99% of the mapped reads (i.e. 337 reads) corresponded to the *Bacillus cereus* group, which is an important causative agent of meningitis.^22^ Nevertheless, we could not confirm whether these bacteria were present and were responsible for the symptoms in each patient. In addition, two bacterial species – *Cutibacterium acnes* (75%) and *Deinococcus proteolyticus* (25%) – were detected in the control sample (Sample #12) of water, although this finding was based on only four reads.

We also conducted bacterial detection using BLASTN^10^ with the following parameters: ‐word_size 9 ‐gapopen 2 ‐gapextend 2 ‐evalue 3.80e‐2. The accuracy of the sequence similarity search using BLASTN is considered to be superior compared to that using minimap2^13^; however, the detected bacterial species using BLASTN were almost identical to those obtained using minimap2, especially for the culture‐positive samples (Samples #1–6; Table 1). In addition, we compared the processing time of minimap2 and BLASTN and found that BLASTN required approximately 5–37.5 times longer processing time compared to minimap2; the processing time increased with the increase in the number of reads (Supplementary figure 1 and Supplementary table 3). These results suggested that the accuracy of a minimap2 search is sufficient to detect bacterial species from 16S rRNA gene amplicon sequencing using MinION, and the time required for bacterial identification is significantly lower than that of BLASTN.

**Figure 3:**
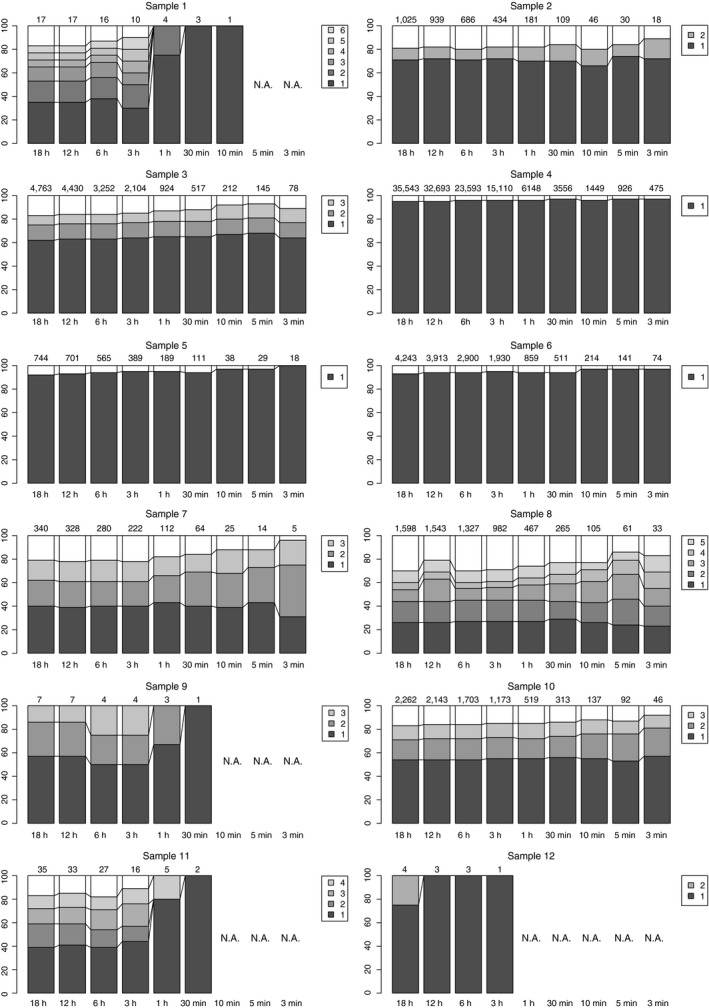
Proportion of matched reads to bacterial species for each sample. Only bacterial species with a match > 10% are shown in the bar graph. The species names are as follows. Sample #1: 1, *Streptococcus mitis*; 2, *Gemella haemolysans*; 3, *Klebsiella pneumoniae* subsp. *pneumoniae*; 4, *Streptococcus pneumoniae*; 5, *Neisseria mucosa*; 6, *Gemmatimonas phototrophica*. Sample #2: 1, *Enterobacter hormaechei* subsp. *steigerwatti*; 2, *Klebsiella pneumoniae* subsp. *pneumoniae*; 3, *Enterobacter hormaechei* subsp. *steigerwatti*; 4, *Acinetobacter indicus*; 5, *Acinetobacter radioresistens*. Sample #3: 1, *Pseudomonas aeruginosa*. Sample #4: 1, *Klebsiella pneumoniae* subsp. *pneumoniae*. Sample #5: *Klebsiella pneumoniae* subsp. *pneumoniae*. Sample #6: 1, *Bacillus thuringiensis*; 2, *Bacillus manliponensis*; 3, *Bacillus anthracis*. Sample #7: 1, *Oerskovia turbata*; 2, *Cellulomonas bogoriensis*; 3, *Cellulosimicrobium cellulans*; 4, *Paraoerskovia marina*; 5, *Cellulomonas gilvus*. Sample #8: 1, *Stenotrophomonas maltophilia*; 2, *Delftia acidovorans*; 3, *Stenotrophomonas chelatiphaga*. Sample #9: 1, *Microbacterium chocolatum*; 2, *Scytonema hofmannii*; 3, *Psychrobacter urativorans*. Sample #10: 1, *Streptococcus pneumoniae*; 2, *Haemophilus influenzae*; 3, *Streptococcus mitis*; 4, *Cutibacterium acnes*. Sample #11: 1, *Cutibacterium acnes*; 2, *Deinococcus proteolyticus*.

We further analysed the time effectiveness of our sequencing analysis. To assess the sequencing time required to identify the causative bacterial species, sequencing data at nine different time points (3 min, 5 min, 10 min, 30 min, 1 h, 3 h, 6 h, 12 h and 18 h) from the beginning of MinION sequencing were compared. The major bacterial species identified at each time point appeared at the first time point in all cases (Figure 3). In particular, major bacterial species were detected within only 3 min of sequencing for Samples #2–8 and #10. Moreover, despite the small number of sequencing reads processed in this short time, the major bacterial species were consistent throughout the entire period (up to 18 h).

**Figure 4:**
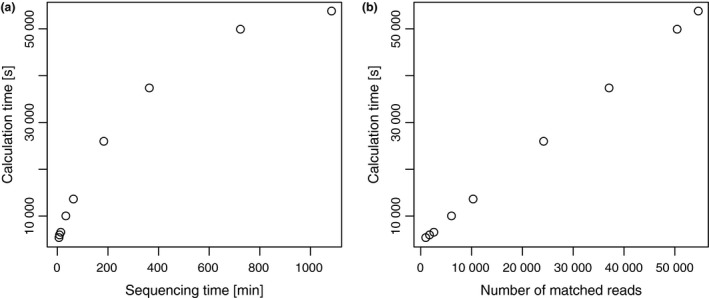
Correlations of sequencing and calculation times. **(a)** Scatter plot of sequencing time (minutes, *x*‐axis) and calculation time (seconds, *y*‐axis). **(b)** Scatter plot of the number of matched reads to the bacterial species genomes (*x*‐axis) and calculation time (seconds, *y*‐axis).

We also determined the time required for processing each calculation step (Table 2 and Supplementary figure 1 and Supplementary tables 1–3). As shown in Table 2, the most time‐consuming step of the computation is the sequence search (Step 3 in Figure 2). The calculation time for the entire computation tended to be longer with more prolonged sequencing (Figure 4a) and strongly correlated with the number of sequencing reads (Figure 4b). This correlation can be explained by the fact that sequence search time depends on the number of query sequences. Therefore, considering the calculation time, it is more advantageous that the number of sequences is smaller; however, the accuracy of species determination gets worse with lower number of sequences.

**Table 2:** Time scale for each data analysis process

Sample	Read count*	Step 1 (s)*	Step 2 (s)*	Step 3 (s)*	Step 4 (s)*	Sum of all steps*
#1	0/19	–/0.0	–/10.4	–/423.7	–/50.6	–/8 min 5 s
#2	18/1,050	0.0/0.8	10.6/10.4	425.4/876.1	50.4/179.8	8 min 6 s/17 min 47 s
#3	78/4,764	0.1/3.9	10.6/10.9	450.6/2930.8	58.4/671.7	8 min 40 s/1 h 17 s
#4	475/35,557	0.4/26.4	10.7/13.0	616.7/19110.5	109.8/4440.9	12 min 17 s/6 h 33 min 11 s
#5	20/774	0.0/0.6	10.6/10.7	424.0/731.2	50.7/126.7	8 min 5 s/14 min 29 s
#6	74/4,259	0.1/3.5	10.8/10.6	448.7/2506.0	54.4/391.8	8 min 34 s/48 min 32 s
#7	5/370	0.0/0.3	10.6/10.4	419.6/576.7	49.0/94.2	7 min 59 s/11 min 22 s
#8	33/1,600	0.0/1.1	10.5/10.8	430.8/1113.6	51.8/234.3	8 min 13 s/22 min 40 s
#9	1/69	0.0/0.1	10.7/10.8	414.8/420.6	47.7/48.8	7 min 53 s/8 min
#10	46/2,270	0.1/1.6	10.6/10.6	434.3/1406.0	53.0/278.5	8 min 18 s/28 min 17 s
#11	603,705	0.1/2.6	10.8/11.1	417.5/446.1	47.9/52.3	7 min 56 s/8 min 32 s
#12	0/5	–/0.0	–/10.7	–/419.3	–/48.6	–/7 min 59 s
SUM	810/54,442	0.84/40.9	106.2/130.4	4482.2/30960.6	572.9/6618.2	1 h 26 min 2 s/10 h 29 min 10 s

* For each step, please see Figure 2. Left and right values indicate the data for 3 min and 18 h of sequencing, respectively.

## Discussion

We detected bacterial species using our updated portable sequencing system from samples of 11 meningitis patients in Zambia. In particular, the sequencing results of four of the six culture‐positive patients were concordant with those of culture‐based methods. Importantly, our sequencing search could detect bacteria at the species levels in a given sample, which cannot be achieved by conventional culture‐based methods. However, such high resolution also comes at a risk of false‐positive detection. For example, *Cutibacterium acnes* was detected as a major bacterium in the water sample (Sample #12) used as a negative control. This bacterial species is commonly found in the human skin^23^, which was also detected from negative control samples (i.e. water) in our previous sequencing analyses.^24^ In addition, as for the other bacterial species found in the Sample #12, *Deinococcus proteolyticus*, these are commonly found from materials, surfaces and dust contaminated by humans and animals as well as soil and sewage.^25^ Therefore, these detected bacterial species in the Sample #12 likely reflect human contamination. The number of reads in the Sample #12 was quite small (four reads), which further suggests a contamination origin during the procedure. However, such DNAs derived from contaminated bacterial species can also be amplified with polymerase chain reaction (PCR)‐based methods. Indeed, in our study, we were not able to quantify the exact amount of DNA owing to the lack of required equipment, which resulted in high variance in the number of reads among samples. Considering our results, even though the number of sequence reads is small, accurate prediction can be made to some extent (Figure 3). Therefore, the results from bacterial identification may not be significantly affected by the uneven reads numbers in multiplexed sequencing in this study.

*Streptococcus pneumoniae, Streptococcus agalactiae *(group B *Streptococcus*), *Neisseria meningitis*, *Haemophilus influenzae* and *Escherichia coli* (particularly the K1 serotype) are currently the most common bacterial pathogens causing acute meningitis in the United States.^16^ However, *Streptococcus, Neisseria* and *Haemophilus* species were found only in the two (i.e. Samples #1 and #10) of the 11 samples analysed in this study. Moreover, no *Escherichia* species were detected in our sequencing analysis. One of the potential reasons for these differences is that the common bacterial pathogens reported are obtained from patients in developed countries. Indeed, in these countries, meningitis is usually observed mainly in elderly people. In this study, we applied samples obtained from patients in Zambia, who have a completely different background from those of developed countries, including a high prevalence of human immunodeficiency virus.^26^ Another possibility is that these common pathogenic bacteria are usually determined by culture‐based methods, which could provide different results from those obtained by sequencing‐based methods in certain cases. Nevertheless, this observation highlights the need to further evaluate the bacterial pathogens causing meningitis in developing countries.

Our current experimental protocol targets the 16S rRNA genes; therefore, other pathogenic agents such as viruses, protists and fungi cannot be detected with the current sequencing‐based method. In addition, it is impossible to determine the drug resistance status of the identified bacteria based on 16S rRNA gene amplicon sequencing. Our computational system itself can potentially be applicable for these purposes since the sequencing data search can be performed at the genomic scale. In addition, recently developed base‐calling software, Guppy, produces accurate sequences with reduced calculation time compared tp Albacore (data not shown). Therefore, the strategy to overcome these challenges could be mainly in the experimental steps. Compared to bacteria, it is generally more difficult to design specific PCR primers for viruses owing to commonly shared genes (such as 16S rRNA genes in bacteria) as well as their rapid mutation rates. Moreover, for eukaryotic species, the potential of host species contamination also needs to be resolved to ensure accurate determination of pathogenic species. These aspects are the next challenges to be tackled for improving sequencing‐based diagnosis of causative agents of infectious diseases.

Studies for sequencing‐based diagnosis of meningitis are ongoing all over the world.^27^, ^28^, ^29^ However, the sequencing methods and bioinformatics pipelines used for the diagnosis are different in different studies. This is because sequencing technology, as well as bioinformatics technology including genome databases, has progressed quickly, making it difficult to establish standards. In addition, sequencing‐based diagnosis usually costs more than conventional culture‐based ones, which makes it difficult to conduct such studies, particularly in developing countries. Indeed, sequencing technology is still being developed; for example, another cheap type flow cell called Flongle was recently released by Oxford Nanopore Technologies, which costs approximately 1/10 the price of the MinION flow cell. The price of sequencing equipment will further reduce with advances in technology.

In conclusion, we tested our improved rapid sequencing diagnosis system based on 16S rRNA amplicon sequencing for the identification of infectious bacterial species of 11 meningitis patients in a medical hospital laboratory in Zambia. As a result, four of the six culture‐positive patients were concordant between sequencing‐based and culture‐based methods; however, for two culture‐positive and five culture‐negative samples, their pathogens were unclear. We found that application of minimap2 reduced the calculation time of species identification without losing its accuracy and that the sequence search time depends on the number of query sequences being processed. The number of sequencing reads required for the rapid diagnosis of infectious bacterial species should be determined depending on the complexity of bacterial species in a sample. For the practical application of sequencing‐based diagnosis of infectious diseases, more examples are required.

## Methods

### Ethical considerations and meningitis patients

The study was approved by the University of Zambia Biomedical Research Ethics Committee (IRB00001131 of IORG0000774), where all experiments were performed. The spinal fluid samples used in this study were collected from 11 meningitis patients at the University Teaching Hospital. The infected bacteria were first identified with conventional culture‐based methods: causative bacteria were identified in six samples, whereas no bacteria were identified in the other five samples. The details of each patient as well as the identified infecting bacteria are summarised in Tables 1 and 3.

**Table 3:** Clinical characteristics of 11 meningitis patients examined in this study

Sample ID	Age/sex	White blood cell count (cells/mL)	PMN%*	Lympho%	Culture results
#1	Unknown/M	0	−	−	+
#2	14 days/M	10	−	−	+
#3	34 years/M	15	−	−	+
#4	13 years/M	0	−	−	+
#5	2 years/F	10	−	−	+
#6	unknown/M	0	−	−	+
#7	32 years/M	500	−	−	−
#8	4 months/F	175	−	−	−
#9	45 years/M	120	98	2	−
#10	unknown/F	200	−	−	−
#11	52 years/F	3520	95	5	−
#12	Water; negative control	NA	−	−	−

* Polymorphonuclear leucocytes.

### DNA extraction, amplicon DNA library preparation and sequencing

DNA was extracted from the cerebrospinal fluid of the 11 meningitis patients using Quick DNA Miniprep kit (Zymo Research, CA, USA). The almost complete full‐length 16S rRNA genes were amplified, and barcode sequences were attached using SQK‐RAB201 Rapid Amplicon Barcoding Kit (Oxford Nanopore Technologies). Based on the manufacturer’s protocol, PCR cycling conditions were as follows: Stage 1, 95°C for 1 min; Stage 2, 25 consecutive cycles of 95°C for 20 s, 55°C for 30 s and 65°C for 2 min; and Stage 3, 65°C for 5 min. The amplicon library was sequenced using the MinION Mk1b sequencer and a FLO‐MIN106.1 flow cell (Oxford Nanopore Technologies). Raw sequencing data (fast5 files) were obtained using the offline version of MinKNOW software ver. 1.10.23 (Oxford Nanopore Technologies). Sequencing data at nine different time points (3 min, 5 min, 10 min, 30 min, 1 h, 3 h, 6 h, 12 h and 18 h) from the beginning of sequencing data were obtained for analysis.

### Data analysis

The data analysis procedure is schematically outlined in Figure 2. For each read (fast5 file), base calling and barcode sorting were performed using Albacore software version 2.1.3 developed by Oxford Nanopore Technologies. First, the fastq files were converted to fasta format using our in‐house script. The simple repetitive sequences were masked using TanTan program^30^ version 13 with default parameters. To remove reads derived from humans, we searched each read against the human genome reference (GRCh38) using minimap2 with default parameters^13^; unmatched reads were regarded as reads derived from bacteria. A total of 5850 representative bacterial genome sequences stored in the GenomeSync database (://genomesync.org) were used for analysis (see Supplementary table 1). For each read, we chose species showing the highest minimap2 score based on alignment length, matched/mismatched sequences and gapped sequences as the existing species in a sample. Taxa were determined using our in‐house script based on the National Center for Biotechnology Information taxonomy database^31^ and visualised using Krona Chart.^32^

### Laptop computers

Two laptop computers were used for the analysis. One was used for MinION sequencing (OS, Windows 10; CPU, Intel Core i7 6700HQ; memory, 8 GB; storage, 960 GB SSD), and the other was used for base‐calling as well as barcode‐sorting, fastq‐to‐fasta conversion, repetitive masking and bacterial identification by Albacore with our in‐house script, TanTan and minimap2, respectively (OS, Ubuntu 16.04; CPU, Intel Core i7 6700K; memory, 32 GB; storage, 1 TB SSD) (Figure 1).

## Author contribution

SI, CS and TI designed the study. SN, KK and TI constructed the system and analysed the data. SI, JY, AO, KH and NA prepared and conducted the experiments. RN, MK, DM and MMM prepared the samples. SN and SI wrote the first draft of the paper. All authors reviewed and approved the final version of the manuscript.

## Conflict of interest

The authors declare no conflict of interest.

## Data Availability

Amplicon sequencing data of the 16S rRNA genes obtained in this study have been deposited in the DDBJ DRA database (https://www.ddbj.nig.ac.jp/dra/index-e.html) under accession numbers DRR172340‐DRR172351.
