# Supplementary material for: Rapid sequencing‐based diagnosis of infectious bacterial species from meningitis patients in Zambia
Source: Clin Transl Immunology. 2019 Nov 5;8(11):e01087. doi: 10.1002/cti2.1087 (PMC6831930; doi:10.1002/cti2.1087)
Supplement: Supplementary file 1 [file CTI2-8-e01087-s001.docx]

**Supplementary figure 1**

Comparison of calculation time by using BLASTN (open square) and minimap2 (open circle). For the details of this figure, please see Figure 4.
